# Inhibiting *N*-acyl-homoserine lactone synthesis and quenching *Pseudomonas* quinolone quorum sensing to attenuate virulence

**DOI:** 10.3389/fmicb.2015.01173

**Published:** 2015-10-19

**Authors:** Kok-Gan Chan, Yi-Chia Liu, Chien-Yi Chang

**Affiliations:** ^1^Division of Genetics and Molecular Biology, Institute of Biological Sciences, Faculty of Science, University of MalayaKuala Lumpur, Malaysia; ^2^Division of Molecular Microbiology, School of Life Sciences, University of DundeeDundee, UK; ^3^Centre for Bacterial Cell Biology, Medical School, Newcastle UniversityNewcastle upon Tyne, UK; ^4^Interdisciplinary Computing and Complex BioSystems Research Group, School of Computing Science, Newcastle UniversityNewcastle upon Tyne, UK

**Keywords:** quorum sensing, quorum quenching, *N*-acyl-homoserine lactone, *Pseudomonas* quinolone signal, *Pseudomonas aeruginosa*, alkylquinolone

## Abstract

Bacteria sense their own population size, tune the expression of responding genes, and behave accordingly to environmental stimuli by secreting signaling molecules. This phenomenon is termed as quorum sensing (QS). By exogenously manipulating the signal transduction bacterial population behaviors could be controlled, which may be done through quorum quenching (QQ). QS related regulatory networks have been proven their involvement in regulating many virulence determinants in pathogenic bacteria in the course of infections. Interfering with QS signaling system could be a novel strategy against bacterial infections and therefore requires more understanding of their fundamental mechanisms. Here we review the development of studies specifically on the inhibition of production of *N*-acyl-homoserine lactone (AHL), a common proteobacterial QS signal. The opportunistic pathogen, *Pseudomonas aeruginosa*, equips the alkylquinolone (AQ)-mediated QS which also plays crucial roles in its pathogenicity. The studies in QQ targeting on AQ are also discussed.

## Introduction

Quorum sensing (QS) is an intercellular communication mechanism of bacteria used to coordinate the activities of individual cells in population level in response to surroundings through production and perception of diffusible signal molecules. The signal synthase, signal receptor, and signal molecules are three essential elements of the basic QS circuit machinery. Genes encoded signal generating proteins are also included among the QS target genes. This forms an autoinduction feedback loop to modulate generation of signal molecules (Fuqua et al., 1994; Ng and Bassler, 2009; Williams and Cámara, 2009). Several bacterial behaviors including virulence factors expression, secondary metabolites production, biofilm formation, motility, and luminescence are regulated by QS (Antunes et al., 2010; LaSarre and Federle, 2013). QS system is part of global regulation networks in bacterial cell related to cyclic-di-GMP second messenger signaling pathways (Srivastava and Waters, 2012), small RNA regulation (Vakulskas et al., 2015), two-components systems (Okkotsu et al., 2014), toxin–antitoxin (Kumar and Engelberg-Kulka, 2014), flagella regulation (Atkinson et al., 2008), and protein secretion systems (Zheng et al., 2010; Atkinson et al., 2011). Through complex regulatory networks bacteria are capable of expressing corresponding genes according to their own population size and of behaving in a coordinated manner.

Bacteria produce ranges of QS signal molecules. *N*-acyl-homoserine lactones (AHLs) are major signal molecules produced by Gram-negative bacteria (Ng and Bassler, 2009; LaSarre and Federle, 2013). Gram-positive bacteria use small linear (e.g., ComX in *Bacillus subtilis*) or circular peptides (e.g., autoinducing peptides AIP in *Staphylococcus aureus*) as signal molecules which are the ligands for the extracellular receptor of a two-component module (Ng and Bassler, 2009). There are other classes of QS molecules including diffusible signaling factor (DSF; *cis*-11-methyl-2-dodecenoic acid) in *Xanthomonas, Xylella, Burkholderia*, and *Pseudomonas* (Deng et al., 2011); α-hydroxyketones (AHKs) in *Legionella pneumophila* and *Vibrio* sp. (Tiaden et al., 2010); furanone molecules (autoinducer-2; AI-2) produced by Gram-negative and -positive bacteria as universal signals for inter- and intra-species communications (Kendall and Sperandio, 2007; LaSarre and Federle, 2013); aromatic molecules (autoinducer-3; AI-3) in enteric pathogens *Salmonella* and *Escherichia coli* O157:H7 served in inter-kingdom communications via the epinephrine signaling system in mammalian cells (Sperandio et al., 2003; Moreira et al., 2010) and alkylquinolone (AQ) in *Pseudomonas* sp. and *Burkholderia* sp (Heeb et al., 2011; Lee and Zhang, 2014). The fragments of peptidoglycan from bacterial cell wall have been suggested as novel QS signals for signaling the growth state of bacteria cell than population size (Dworkin, 2014).

Due to its key roles in bacterial population behaviors and pathogenicity, QS has been suggested to be the target for novel bacterial infection therapy (Zhang and Dong, 2004; Rasmussen, 2006; LaSarre and Federle, 2013). By reducing concentration of signals or interrupting the interactions of signal on receptor protein, the expression of QS-regulated genes can be disturbed and bacterial virulence can be subsequently attenuated (Dong et al., 2000, 2001). These approaches coined as quorum quenching (QQ) were considered as alternatives against bacterial infections (Cámara et al., 2002; Zhang and Dong, 2004; González and Keshavan, 2006). Several natural compounds and enzymes from prokaryotic (Dong et al., 2000; Wang, 2004; Chu et al., 2013) and eukaryotic organisms including plants (Vandeputte et al., 2011; Koh et al., 2013), murine (Yang et al., 2005), and human (Chun et al., 2004; Ozer et al., 2006) have been discovered and shown the capability of inhibiting bacterial QS systems. Broad advances on QQ studies and various types of QS inhibitors are also highlighted in several reviews (Chun et al., 2004; Yang et al., 2005; Ozer et al., 2006; Dong et al., 2007; Chu et al., 2013; Kalia, 2013; Koh et al., 2013; LaSarre and Federle, 2013; Nazzaro et al., 2013). Here we review the development of inhibiting of AHL synthesis. In the genome of *Pseudomonas aeruginosa*, an opportunistic human pathogen, QS is responsible for the regulation of around 10% of genes (Williams and Cámara, 2009). AQ–QS coupled with AHL-QS play crucial roles in virulence regulation. Thus the recent QQ studies on AQ–QS are also discussed.

## AHL Synthesis and Inhibition

More than 30 different AHLs have been identified. AHLs generally consist of a homoserine lactone ring and of a fatty acyl side chain ranging from 4 to 18 carbons (Chhabra et al., 2005). The regulation of bioluminescence in *Aliivibrio fischeri* (former *Vibrio fischeri*) (Urbanczyk et al., 2007) is the archetypal example of AHL-QS. The genes for bioluminescence are encoded in an operon (*luxICDABE*) where *luxI* encodes the AHL synthase that generates *N*-(3-oxo-hexanoyl)-L-homoserine lactone (3-oxo-C6 HSL) and *luxCDABE* encode for proteins responsible for bioluminescence production. This operon is regulated by the LuxR protein, a transcriptional regulator. Binding of 3-oxo-C6-HSL by LuxR activates the expression of the *luxICDABE* operon which in turns results in the production of more of this signal molecule, through an autoinduction loop, and bioluminescence (Fuqua and Greenberg, 2002). *S*-adenosylmethionine (SAM) is the main amino acid substrate for the homoserine lactone ring, while the acyl chain of AHL is derived from a 6-carbon acyl-ACP (acyl carrier protein). The LuxI catalyzes the formation of an amide bond between the two substrates. SAM binds to the active site of LuxI, and then the acyl group from a specific acyl-ACP is transferred to the methionine on SAM and forms an amide bond. Lactonization of the intermediate results in the production of AHL and methylthioadenosine (MAT; Parsek et al., 1999). Two regions of LuxI are essential for the full function of the enzyme. Residues 25 to 104 are thought to be the region of the enzymatic catalysis, while residue 133 to 164 are involved in the selection of the appropriate acyl-ACP substrates (Hanzelka and Greenberg, 1996).

Homologs of *luxI*/*luxR* are involved in Gram-negative bacteria (Miller and Bassler, 2001; LaSarre and Federle, 2013). AHL synthases from more than 40 different bacteria share four conserved regions in their protein sequences and within them eight residues are completely conserved (Fuqua et al., 1994). The X-ray crystallography study of an AHL synthase EsaI, a LuxI-type protein producing 3-oxo-C6-HSL from *Pantoea stewartii*, has been revealed (Watson et al., 2002). A model for catalyzing the amide bond between the two substrates based on this structural study has been proposed. Many of the conserved residues across LuxI-type synthases lie on the same face of the EsaI and are localized in the active site in N-terminus. This supports a notion that SAM and acyl-ACP interact with this region. The presence or absence of a serine/threonine at residue 140 in AHL synthase may be crucial for selecting different acyl-ACP substrates. LasI, EsaI, and LuxI which mainly produce 3-oxo-AHLs have a conserved threonine at this position whilst the absence of threonine at position 140 in RhlI, CerI, SwrI, and AsaI seems to preferentially produce AHLs lacking 3-oxo or 3-hydroy moiety (Watson et al., 2002). The crystal structure of LasI, the synthase from *P. aeruginosa* mainly produces *N*-(3-oxododecanoyl)-homoserine lactone (3-oxo-C12-HSL), further reveals the mechanism of AHL synthesis. The conserved residues in LasI N-terminus form a binding packet for SAM and the structure of the acyl-ACP binding packet is formed as a tunnel which allow the binding with different acyl-ACPs with longer side chain in contrast to the restrictive hydrophobic packet in EsaI (Gould et al., 2004). The acceptance of various acyl-ACPs in LasI may explain the wild spectrum of AHL production profile in *P. aeruginosa* (Ortori et al., 2011).

*N*-acyl-homoserine lactone molecules act not only in bacterial communication but also in cross-kingdom conversation with eukaryotic cells (Joint et al., 2002; Twigg et al., 2013). AHLs modulate the behaviors of innate immune cells and interfere with the signaling pathways in epithelial cells (Holm and Vikström, 2014). Inhibition of AHLs production is the strategy to reduce the AHLs-mediated virulence factors and to prevent AHLs-promoted tissue damages and inflammation. To study the kinetic process of AHLs synthesis by RhlI from *P. aeruginosa*, the end products MTA and several substrate analogs including holo-ACP, sinefungin, D/L-*S*-adenosylhomocysteine, L-*S*-adenosylcysteine, and butyryl-SAM have been tested for their inhibition effects on AHLs production *in vitro* (Parsek et al., 1999). However their effects on QS and QS-related phenotypes *in vivo* are unclear (Rasmussen, 2006). More inhibitors targeting on the AHL synthesis precursors or their synthesis have been identified. A small molecule, triclosan, reduces AHL synthesis by inhibiting the precursor production from enoyl-ACP reductase (Hoang and Schweizer, 1999). The 5-MAT/*S*-adenosyl-homocysteine nucleosidase (MTAN), which plays crucial part in both AHL and AI-2 synthesis, was inhibited by immucillin A (ImmA) derivatives and DADMe-ImmA derivatives (Singh et al., 2005a,b, 2006). These inhibitors not only show the effective potency on AHL synthesis but also inhibit the central amino acid and fatty acid metabolisms by which may affect other cellular functions. This imposes selective pressure to bacterial cells and increases the risk of resistance. For example *P. aeruginosa* develops resistance to triclosan because of active eﬄux pumps (Schweizer, 2003). Thus, it is necessary to identify inhibitors specifically targeting on AHLs synthesis without interrupting metabolisms.

In *Burkholderia glumae*, the pathogen of rice grain rot, virulence factor biosynthesis and transportation, protein secretion and motility are controlled by *N*-octanoyl-HSL (C8-HSL) produced by TofI. Two acyl-HSL analogs have been identified to inhibit the C8-HSL mediated QS. One of the inhibitor, E9C-3oxoC6, competitively inhibits C8-HSL binding to its cognate regulator, TolR. The other inhibitor, J8-C8 (*N*-3-oxocyclohex-1-enyl octanamide), inhibits C8-HSL synthesis in a dose-dependent manner. Crystal structure analysis suggested that the TofI, which is similar to its homologues, LasI and EsaI, consists of two substrate binding sites (Watson et al., 2002; Gould et al., 2004; Chung et al., 2011). A putative pocket in apo-TofI structure has been identified which is bound by J8-C8. This study suggested J8-C8 occupies the acyl-ACP substrate-binding site of TofI and inhibits its function (Chung et al., 2011).

Besides rationally designed analogs, natural products isolated from various sources also exhibit QQ properties (Koh et al., 2013). Three pure botanic compounds, salicylic acid, tannic acid, and *trans*-cinnamaldehyde, showed the inhibition on *P. aeruginosa* AHL synthase. Further liquid chromatography–mass spectrometry (LC–MS) analysis suggested the *trans*-cinnamaldehyde specifically targets short-chain AHL synthase RhlI. The RhlI/R-QS in *P. aeruginosa* is involved in the production of pyocyanin, a green phenazine pigment functioning as a toxin and promoting biofilm formation. The *trans*-cinnamaldehyde inhibits pyocyanin production in a dose-dependent manner. Successful molecule docking suggested *trans*-cinnamaldehyde occupies the SAM substrate binding sites of RhlI counterpart, LasI in *P. aeruginosa* and the acyl-chain substrate binding site of EasI in *Pa. stewartii* (Chang et al., 2014). Furthermore cinnamaldehyde and its derivatives target on AI-2 based LuxR in *Vibrio* sp. and reduce the LuxR DNA binding ability which lead to the reduction of biofilm formation, sensitivity to starvation and antibiotics treatment, reduction of pigment and virulence factors production and attenuated pathogenicity (Brackman et al., 2008, 2011). These studies suggested the potentiality of cinnamaldehyde as a QQ compound against bacteria infections.

## Quenching AQ-Mediated QS

A non-AHL signaling molecule produced by *P. aeruginosa* was described and termed as PQS (*Pseudomonas* quinolone signal) by Pesci et al. (1999). This molecule was chemically characterized as 2-heptyl-3-hydroxy-4-(1H)-quinolone, part of the 4-hydroxy-2-alkyl quinoline series (HAQ; Pesci et al., 1999). Other major molecules comprised the C7 and C9 long alkyl chain including 2-heptyl-4-quinolone (HHQ), 2-nonyl-4-quinolone (NHQ), 2-heptyl-4-quinolone N-oxide (HHQNO), 2-heptyl-4-hydroxyquinoline N-oxide (HQNO) and 2-nonyl-4-hydroxyquinoline N-oxide (NQNO) are also produced by *P. aeruginosa*. More than 50 different AQs have been found in *P. aeruginosa.* These molecules all belong to the family of 2-alkyl-4-quinolones (AQs) which have been previously studied for their antimicrobial properties (Heeb et al., 2011; Ortori et al., 2011).

2-heptyl-4-quinolone and *Pseudomonas* quinolone signal are the major AQ–QS signaling molecules in *P. aeruginosa*. The AQ–QS system consists of multiple genes. The *pqsABCDE* in an operon are essential for AQs synthesis. PqsA catalyzes the conversion from anthranilic acid produced by *phnAB*, which are adjacent to *pqsABCDE* operon, to anthraniloyl-coenzyme A (CoA). PqsD then condenses anthraniloyl moiety from anthraniloyl-CoA with malonyl-CoA to form intermediate 2-aminobenzoyl-acetyl-CoA (2-ABA-CoA) for subsequently synthesizing 2,4-dihydroxyquinolone (DHQ) with unknown function or 2-aminobenzoylacetate (2-ABA). The decarboxylating coupling of 2-ABA to an octanoate group linked to PqsBC produces HHQ (Bera et al., 2009; Dulcey et al., 2013). HHQ acts as a signaling molecule or can then be transformed to PQS by the mono-oxygenase, PqsH (Pesci et al., 1999; Diggle et al., 2006). PqsE is required for *P. aeruginosa* virulence in plant and animals infection models and biofilm formation (Rampioni et al., 2010). A recent study suggested PqsE is also involved in the HHQ synthesis as a thioesterase, hydrolyzing the 2-ABA-CoA to form 2-ABA. Although PqsE plays crucial role in *P. aeruginosa* pathogenicity, its catalytic role in AQ biosynthesis can be replaced by a broad specificity thioesterase, TesB (Drees and Fetzner, 2015). Two other genes, *pqsH* and *pqsL*, involved in AQ synthesis are located separately elsewhere on the chromosome in *P. aeruginosa*. PqsH, FAD-dependent monooxygenase, is required for the conversion of HHQ into PQS. PqsL is also a monooxygenase which is required for HQNO synthesis (Heeb et al., 2011). PQS or HHQ binds to and activates the LysR-type transcriptional regulator PqsR (also known as MvfR), which in turn induces the expression of the *pqsABCDE* operon and possibly the *phnAB* operon and triggers the typical QS autoinducing response enhancing AQ biosynthesis (Maddocks and Oyston, 2008; Heeb et al., 2011).

Alkylquinolone–quorum sensing cooperates with the AHL-QS systems, *lasI/R* and *rhlI/R*, in *P. aeruginosa* (Xiao et al., 2006; Heeb et al., 2011). PQS has been shown to regulate the expression of the AHL synthase gene *rhlI* (Pesci et al., 1999; McKnight et al., 2000). Furthermore, it has been demonstrated that PQS is essential for the activation of certain *rhl*-dependent genes (Diggle et al., 2003). The *las* QS system increases the expression of *pqsR* and *pqsA* as well as controlling the expression of *pqsH* indicating that *las* acts as a positive regulator of PQS (Déziel et al., 2004; Xiao et al., 2006). However, the biosynthesis of PQS only partially relies on the *las* system since PQS is still produced in the absence of *lasR* (Diggle et al., 2003). In contrast to the *las* QS system, the *rhl* system negatively regulates the PQS production (Xiao et al., 2006; Heeb et al., 2011).

Alkylquinolone–quorum sensing also plays important roles in pathogenicity. Mutants on AQ–QS showed reduced *P. aeruginosa* virulence in infection models (Cao et al., 2001; Diggle et al., 2003; Déziel et al., 2004). These results suggest that PQS or HHQ signaling pathway could be a novel target against *P. aeruginosa* infecyions. A sesquiterpene, farnesol produced by the fungus *Candida albicans*, decreases the level of *pqsA* expression by interfering with PqsR-mediated transcription activation and sequentially reduces the PQS and PQS-regulated pyocyanin production. However, in the PQS-defective *lasR* mutant farnesol restores PQS production by inducing PqsH via RhlI/C4-HSL activation (Cugini et al., 2007, 2010). Around 50% of strains isolated from lungs of late stage cystic fibrosis (CF) patients are deficient in *lasR* function (Winstanley and Fothergill, 2009). The fungal–bacterial communication via farnesol is still unclear and may provide a new target for mitigating bacterial chronic infection in CF lungs.

A monomeric enzyme, Hod (3-hydroxy-2-methyl-4(1*H*)-quinolone 2,4-dioxygenase) from *Arthrobacter* sp. strain Rue61a involved in the degradation of 2-methylquinoline (quinaldine), is able to cleave PQS to *N*-octanoylanthranilic acid and carbon monoxide but not HHQ. Exogenous Hod reduces the PQS production and PQS-regulated virulence factors, lectin A, pyrocyanin, and rhamnolipids in *P. aeruginosa*. Hod also attenuates the pathogenicity of *P. aeruginosa* in a plant leaf infection model (Pustelny et al., 2009). It has been reported that, a group of analogs of PQS precursor anthranilatic acid (AA) including methylanthranilate (MA), 2-amino-6-fluorobenzoic acid (6FABA), 2-amino-6-chlorobenzoic acid (6CABA), and 2-amino-4-chlor-obenzoic acid (4CABA), acts as competitors for PqsA active site and as inhibitors for HHQ and PQS production resulting in reduced pathogenicity of *P. aeruginosa* in mice infection models (Calfee et al., 2001; Lesic et al., 2007). Recently Soh et al. (2015) reports an environmental bacterium, *Achromobacter xylosoxidans* Q19 from rainforest soil, is capable of oxidizing PQS into 2-heptyl-2-hydroxy-1,2-dihydroquinoline-3,4-dione (HHQD), which was elucidated by mass spectrometry and nuclear magnetic resonance spectroscopy. The PQS oxidation to HHQD also occurs with less efficiency in the *A. xylosoxidans* and *P. aeruginosa* from CF lung which suggests that the hydroxylated PQS is a common molecule in soil and in CF lungs (Soh et al., 2015). However, the biological function of HHQD is still unknown.

Besides targeting on PQS molecules production or modification, a group of compounds with a benzamide-benzimidazole backbone targeting on MvfR (PqsR)-regulated pathways have been identified by using a whole-cell high-throughput screen (HTS) and structure–activity relationship (SAR) analysis. These compounds inhibit the production of pyocyanin in several clinically isolated *P. aeruginosa* strains; limit the formation of antibiotic-resistant cells; rescue mice macrophage from the bacterial cytotoxicity; attenuate the bacterial pathogenicity in acute thermal injury and lung infection murine models; reduce the accumulation of macrophage at the infection sites resulting in inhibition of inflammation evoked by bacteria and inhibit bacterial persistence for developing chronic infection in burned mice. One of the most effective compounds, M64 molecule, binds to MvfR and decreases the MvfR–DNA binding affinity by 10-folds which results in the reduction of MvfR-activated virulence factors. These compounds have been suggested as next generation therapeutic agents against bacterial infections (Starkey et al., 2014).

## Conclusion

Here we summarize recent studies of AHL synthesis inhibition and QQ on *P. aeruginosa* AQ–QS (**Table 1**). Although QS is an ideal target to attenuate bacterial virulence and pathogenicity, several studies warn that bacteria rapidly evolve and spread the resistance against QS inhibitors like the fate of antibiotics (García-Contreras et al., 2013; Kalia et al., 2013). Furthermore Decho et al. (2010) suggested that the biologically or chemically modified signals might interact with unexpected signal receptors for unpredicted outcomes in the complex natural environments. Little is known about the effects of QS inhibitors or enzymes on the broader microorganisms’ communities or the fate of products from signal molecules degradation and modifications in the environments (Decho et al., 2010). Many questions remain for further investigation. Thus it is highly desired to further investigate the virulent roles of QS signals in infections and to explore the strategy to eliminate their production rather than to modify or to degrade them for diminishing unpredicted impacts to environments.

**Table 1:** Overview of inhibitors on *N*-acyl-homoserine lactone (AHL) synthesis and on alkylquinolone (AQ)-mediated quorum sensing (QS) system.

Molecules/enzymes	Mechanism of inhibition	Reference
**Inhibitors of AHL synthesis**		
MTA and substrate analogs: holo-ACP, sinefungin, D/L-*S*-adenosylhomocysteine, L-*S*-adenosylcysteine, and butyryl-SAM	Potentially occupying the substrates binding site of RhlI in *Pseudomonas aeruginosa*	Parsek et al., 1999
Triclosan	Inhibiting the AHLs precursor production from enoyl-ACP reductase	Hoang and Schweizer, 1999
Immucillin A (ImmA) derivatives and DADMe-ImmA derivatives	Inhibiting MTAN, the enzyme involved in MTA depurination	Singh et al., 2005a,b, 2006
J8-C8	Occupying the acyl-ACP substrate-binding site of TofI in *Burkholderia glumae*	Chung et al., 2011
*Trans*-cinnamaldehyde	Potentially binding to substrate binding sites of RhlI in *P. aeruginosa*	Chang et al., 2014
**Inhibitors of AQ-based QS**		
Analogs of 2-heptyl-4-quinolone (HHQ) and PQS (*Pseudomonas* quinolone signal) precursor anthranilatic acid (AA): methylanthranilate (MA), 2-amino-6-fluorobenzoic acid (6FABA), 2-amino-6-chlorobenzoic acid (6CABA), and 2-amino-4-chlor-obenzoic acid (4CABA)	Potentially competing for activate site of PqsA in *P. aeruginosa*	Calfee et al., 2001; Lesic et al., 2007
A group of compounds with a benzamide–benzimidazole backbone	Binding to MvfR (PqsR) and reducing MvfR DNA binding activity	Starkey et al., 2014
Farnesol	Decreasing the level of *pqsA* expression	Cugini et al., 2007
Hod (3-hydroxy-2-methyl-4(1*H*)-quinolone 2,4-dioxygenase)	Cleaving PQS to *N*-octanoylanthranilic acid and carbon monoxide	Pustelny et al., 2009
Unknow enzyme from *Achromobacter xylosoxidans* Q19	Oxidizing PQS into 2-heptyl-2-hydroxy-1,2-dihydroquinoline-3,4-dione (HHQD)	Soh et al., 2015

## Conflict of Interest Statement

The authors declare that the research was conducted in the absence of any commercial or financial relationships that could be construed as a potential conflict of interest.
